# Dynamic Interaction between Student Learning Behaviour and Learning Environment: Meta-Analysis of Student Engagement and Its Influencing Factors

**DOI:** 10.3390/bs13010059

**Published:** 2023-01-09

**Authors:** Jian Li, Eryong Xue

**Affiliations:** China Institute of Education Policy, Faculty of Education, Beijing Normal University, Beijing 100875, China

**Keywords:** student engagement, systematic review, meta-analysis, higher education, international higher education

## Abstract

Student engagement plays a significant role in promoting student learning outcomes in the higher education context. The goal of this meta-analysis was to investigate factors influencing student engagement in higher education institutions in different contexts. The meta-analysis integrated data from 93,188 participants and 148 effects across studies to address this issue. The meta-analysis revealed 14 factors affecting students’ learning participation. The classification was based on internal and external factors, aiming to explore the main factors influencing students’ intention, behavior, and process of learning participation. The external influencing factors with moderate correlations were the teacher-student relationship (R = 0.456, *p* < 0.001) and positive teacher behavior (R = 0.419, *p* < 0.001). Additionally, the main external influencing factors were partnership (R = 0.174, *p* < 0.001), environmental support (R = −0.028), negative teacher behavior (R = −0.064), and negative learning behavior (R = −0145), which were all negatively correlated with learning participation. The results also indicated that factors influencing student engagement can be divided into two categories: promoting factors and hindering factors. The promoting factors include students’ positive emotion, positive learning behavior, positive teacher behavior, the teacher-student relationship and partnership, students’ learning and thinking ability, the support of learning resources, students’ individual and personality characteristics, and teaching factors. The hindering factors include lack of environmental support, negative student behavior, and negative teacher behavior. Further, conceptual and practical implications are discussed in relation to these findings.

## 1. Introduction

Student engagement plays an important role in shaping educational achievement in the higher education system, and factors influencing student engagement may have profound and long-lasting implications for student performance and learning outcomes. It is necessary to talk about how to promote student engagement in the current higher education system worldwide. It is considered to be of pressing interest, and there is a need for researchers to analyze this topic using meta-analysis. Several studies have examined the factors that impact student engagement and learning outcomes because it is clear that student engagement plays a key role in positive student development in post-secondary education [1]. 

Student engagement is a complex concept, and scholars have offered many perspectives on how to examine and conceptualize it. Zepke (2018) defined “student engagement” as a construct used to identify what students do, think about, and feel when learning, and how teachers can improve that doing, thinking, and feeling in instructional settings. The critique, learning agency/democracy, purposes of learning, knowledge, and values should be considered to fully understand the complexity of student engagement [2]. 

In addition, fewer studies have explored factors influencing student engagement in higher education institutions in different contexts. Thus, in this study, an investigation was conducted to understand factors influencing student engagement in the educational context. In applying a meta-analytic approach, we estimated the average association between influencing factors (e.g., positive/negative learning perception, learning experience, peer interaction, student-teacher relations, and supportive learning environment) and student engagement/involvement. The relationship between learning outcomes and student engagement and how report-level characteristics (e.g., publication type) might moderate this relationship were also examined. Along with the analyses, we also discuss the limitations of existing studies in this field and make suggestions for future work.

## 2. Literature Review

The studies in the field of student engagement have been increasing over recent decades [3], and the number of reports included in the present study is comparable with that of recently published meta-analyses on student engagement and student learning communities [4,5,6]. Additionally, many more studies on topics such as peer interaction, student-teacher relations, and supportive learning environments are likely to take place in the next few decades. A systematic meta-analytic review of the scope of emerging research can effectively influence future study. Further, through our review, we aim to guide the field of student engagement in promising directions (both methodologically and theoretically) to avoid too many unnecessary detours.

### 2.1. Student Engagement and Learning Performance

Student engagement is fundamental to learning performance and plays a pivotal role in student success [7]. Tinto (2014) noted that student engagement is complex and can be viewed from multiple perspectives, with both institutional and structural factors closely interacting. Kahu and Nelson (2018) examined the framework of student engagement through a cultural lens to introduce an educational interface for individual psychosocial development related to both institutional and personal elements. This educational interface is proposed as a tangible approach to understanding the complicated interaction between students and institutions and how these interactions influence learning engagement. Henrie et al. (2015) examined longitudinal measures of student engagement in blended learning. The relationship between student engagement and course levels was investigated to determine the aspects influencing student engagement in a blended course, arguing that the clarity of instruction and relevance of activities influenced student satisfaction more than the medium of instruction.

Student engagement is associated with learning tools and practices to ensure a successful learning experience. Tan and Hew (2016) examined how the use of meaningful gamification affects student learning, engagement, and affective outcomes in a blended learning Research Methods class using a combination of experimental and qualitative approaches. The students in that study strongly agreed that blended learning has a positive impact on their learning engagement.

### 2.2. Factors Influencing Student Engagement

Several studies have been conducted to determine factors influencing student engagement, and student engagement has been well-studied in a range of contexts, focusing on the cognitive, emotional, and behavioral integration of students’ learning performance [8,9]. Kabu (2013) argued that students’ characteristics and experiences influence their learning engagement. The learning community promotes student engagement, learning outcomes, and overall satisfaction with post-secondary education [10,11,12]. Participation in the learning community is connected to success, including learning outcomes, academic and social experiences, and positive attitudes toward campus life. Student engagement mediates the relations between personal resources and observed learning behaviors, and learning resources are indirectly and positively connected to learning activities through student engagement [13].

Student engagement involves three main factors: behavioral, emotional, and cognitive factors. The behavioral factors include effort, persistence, concentration, asking questions, and class communication [14,15]. The emotional factors involve students’ affective communication and practices on campus [16]. The cognitive factors focus on psychological involvement through learning, understanding, and mastering the knowledge and skills taught in schools [17]. Boatman and Long (2016) investigated the relationship between financial aid impact and college student engagement by analyzing the Gates Millennium Scholarship (GMS) Program. It was found that the program positively influences students’ academic and social engagement behaviors. For example, gamification also plays an important role in influencing student engagement. It also affects students’ health. When the game players put a lot of energy into playing games, nature, in addition to making it basically impossible to do other things, has brought a lot of adverse effects, such as impaired eyesight, physical decline, and irregular diets caused by digestive system diseases. Sitting in front of a computer for a long time can lead to neurological disorders, an imbalance of hormone levels in the body, reduced immune function, and a variety of other medical conditions. It affects students’ normal study and life and also has violent tendencies [18].

Carini et al. (2006) examined the relationship between student engagement and learning. Student engagement was associated with desirable learning outcomes, such as critical thinking and positive grades. The impact of student engagement, student satisfaction, and perceived learning in online learning environments was investigated further by Gray and DiLoreto (2016). They argued that student engagement plays an important role in the relationship between learner interaction and instructor presence in terms of both perceived student learning and student satisfaction. Gunuc and Kuzu (2015) contend that student engagement includes a sense of belonging and valuing, cognitive, emotional, and behavioral engagement, peer relationships, and relationships with faculty members. Ultimately, student engagement promotes learning quality and performance. 

The dimensions of student engagement are divided into two aspects: campus engagement (valuing, sense of belonging, and participation) and class engagement (cognitive, emotional, and behavioral engagement) [19,20]. They examined the relationship between student engagement and retention for community college students. It was found that student engagement had a positive impact on student learning communities, influencing student grades and course completion rates. The higher levels of student engagement resulted in lower absenteeism in the learning community. Thus, student engagement is associated with established learning communities and short-term student success.

Teacher support plays a positive role in influencing student engagement and learning outcomes [21]. The professional learning communities of students and teachers offer personalized learning environments. Kuh et al. (2008) suggested that student engagement has a positive impact on first-year college grades and students’ learning persistence. The benchmarks of student engagement consider levels of academic challenges, student-faculty interaction, educational experiences, collaborative learning, and supportive campus environments [22]. In addition, building a sense of belonging is linked to enhanced student engagement through students’ experiences of participating and learning. The students’ sense of belonging interacts with their engagement and retention [23].

Furthermore, cultivating a sense of belonging, building students’ learning identities, and making sense of their performance contribute to positive peer communities and engagement. Quin (2017) observed a contextual connection between teacher-student relationships and student engagement from a systematic perspective. The multiple indicators of student engagement used in that study included psychological engagement, academic grades, school attendance, disruptive behaviors, suspension, and dropout [24]. Shernoff et al. (2016) observed that student engagement is a function of environmental complexity in high school classrooms. The quality of the learning environment is associated with the quality of the classroom experience. It was found that environmental complexity predicted student engagement and a sense of self-esteem in the classroom. Additionally, You (2016) argued that psychological capital plays a positive role in promoting learning empowerment and student engagement, and Shernoff et al. (2016) found that blended learning environments have a particularly important impact on learning performance and engagement. The “flipped classroom” pedagogy is also considered beneficial for improving student engagement and learning outcomes [25]. 

**Figure 1:**
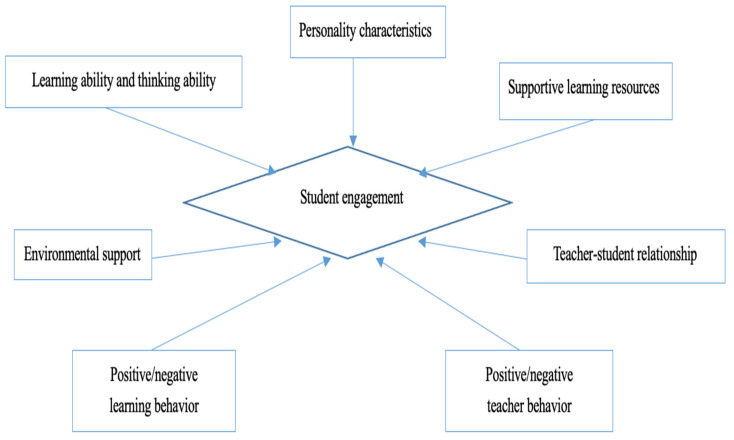
Factors influencing student engagement.

The literature, thus, suggests several key influencing factors on student engagement, including students’ learning and thinking abilities, behavior, and individual personality characteristics; teachers’ behavior, the teacher-student relationship; support through learning resources; and environmental support. These internal and external factors contribute to understanding student engagement in context and are summarized in Figure 1. In Figure 1, based on the current literature review, we can conclude several factors influence students’ engagement, including their learning and thinking abilities, behavior, and individual personality characteristics: teachers’ behavior in the teacher-student relationship, support through learning resources, and environmental support. All these seven influencing factors contribute to shaping student engagement accordingly. 

## 3. Methods

The Meta-analyses checklist was used for the meta-analysis undertaken in this study. The coding sheet included all the reported effect sizes, moderating variables, and analysis scripts, which can be examined in the supplemental materials. The articles were retrieved by a computerized literature search of the online databases at the Education Resources Information Center (ERIC), EBSCO, Springer, and the Web of Science. This literature collection was analyzed for studies published up to December 2021 with three categories of key phrases used to search key words containing variables concerning student engagement or student involvement (student engagement*, student involvement*, or attachment* participants).

### 3.1. Selected Benchmarks and Data Resources

The reason we choose meta-analysis is that the meta-analysis statistical method is the re-statistics of many existing empirical literatures. The statistical indicators in relevant literature are analyzed again by using corresponding statistical formulas to analyze the real correlation between two variables according to the statistical significance obtained. Along with the nature of the meta-analysis statistical method, we aim to explore the existing empirical studies on student engagement worldwide to examine the real connections among various factors. In addition, according to Glass, a meta-analysis is an analysis of an analysis with the following main characteristics: Meta-analysis is a quantitative analysis method, that involves not the statistics of original data but the re-statistics of statistical results. The meta-analysis should include studies of different qualities; the meta-analysis seeks a comprehensive conclusion. This study follows the key principles above to explore student engagement and its influencing factors. The selected source documents were based on the Education Resources Information Center (ERIC), EBSCO, Springer database, and the Web of Science, and they were filtered following several steps, including identifying the research questions, collection and screening, content extraction, total effect analysis, specific effect analysis, and interpreting the results. 

In addition, several studies were included in the meta-analysis when they met benchmarks. This research aimed to evaluate the relations between any dimensions of student engagement (e.g., student participation, learning communities) or the engagement-performance relationship (e.g., learning outcome, academic persistence). Thus, the study had to report on student engagement or involvement or interrelated concepts such as collaborative learning, student-faculty interaction, learning experience, and peer interaction. We were interested in the general population, but most research has concentrated on institutional-based samples where learning groups may influence the magnitude or direction of effect sizes. The mean age of the participants had to fall within the age range of the schooling period used in the current study, that is, the 18–35-year range, which is commonly considered for students in tertiary education. The research had to be published in English in a peer-reviewed journal, with the full text available for download. Further, we did not select unpublished work, review articles, book chapters, dissertations/theses, or conference papers if these duplicate papers had already been published in a peer-reviewed journal. The peer-reviewed publications included in this study may also have been included in prior meta-analyses. The meta-analysis included unpublished studies to enable a search for evidence of publication bias. 

### 3.2. Selection Procedure

**Figure 2:**
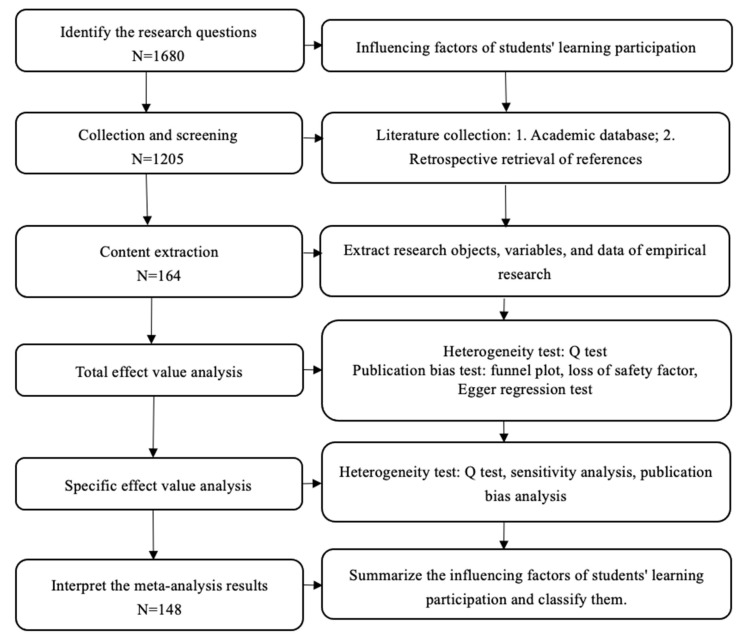
Preferred Reporting Items for Systematic Reviews and Meta-Analyses (PRISMA) flowchart used to identify studies for detailed analysis of student engagement and learning outcomes.

Our initial search turned up 1680 potentially relevant publications. These were carefully screened to determine whether they met the inclusion benchmarks. Several publications were excluded because they did not include an appropriate measure of student engagement/student involvement. The corresponding authors of the selected articles were contacted by email to request additional information. Several authors declined our invitation because they no longer had access to the data (8%); some could not be contacted because no valid e-mail address was found (19%); and others provided us with the necessary correlations (15%), yielding 12 additional articles to include. However, most of our e-mails remained unanswered (72%). Finally, 148 studies met the selection benchmarks and were included in the meta-analysis. The PRISMA flowchart shown in Figure 2 depicts the full search and inclusion process. 

### 3.3. Coding and Sampling

A detailed coding scheme was generated along with guidelines for recording study descriptors and features potentially moderating the relationships between student engagement and learning performance in education. The research descriptors contained the general information for all research, including the co-authors’ names, year of publication, title of publication, descriptive information on data collection, and sample size. The research characteristics moderating the connections between student engagement and learning outcome in the educational context were grouped into two moderator categories: moderators of theoretical interest and methodological attributes.

Theoretical moderators: In order to assess possible moderating effects of different student engagement types, we divided student engagement practices and characteristics into three categories: emotional engagement, including student-teacher relations, peer interaction, and positive/negative feelings; behavioral engagement, including positive/negative learning behaviors; and cognitive engagement, including learning characteristics, learning capacities, and supportive learning attributes. The research was coded as follows: 1 = emotional engagement, 2 = behavioral engagement, and 3 = cognitive engagement. Table 1 provides a detailed overview of the subdivision of student engagement dimensions by age range and cultural identities. 

The ages were coded consistently. In cases where studies did not report age but rather school grade, we used the average age of that grade. For example, children in 7th grade in the United States are, on average, between the ages of 12 and 13 years, and we therefore considered 2.5 years the mean age for the sample. In addition, individualism and ethnicity were considered in describing cultural identification. The level of individualism of the institutions in which the data were collected was coded according to Hofstede’s individualism score (www.hofstede-insights.com, accessed on 20 April 2021). The score is a continuous index, with higher scores for more individualistic societies and lower scores for more collectivistic ones. The ethnicity of students in the sample was coded as follows: 1 = balanced (i.e., no ethnicity; more than 54% of the sample); 2 = > 62% White; 3 = > 66% African or African American; 4 = > 54% Asian or Asian American; 5 = > 60% Hispanic; or 6 = other. The student gender was categorically coded based on the percentage of men and women in the sample as 1 = overall balanced (the percentage of men and women in the sample ranged between 40% and 60%); 2 = > 60% men, or 3 = > 60% women.

The analysis required the inclusion of effect sizes from each study while consistently taking dependency into consideration. The categories of positive/negative engagement and learning performance relationships were not mutually exclusive, along with the effect sizes of various categories. For longitudinal research designs, studies focused on several ages, and in some cases, gender was considered separately in terms of the effects of student engagement on learning outcomes. The multiple analyses helped us decrease the bias in the studies. The research was coded for the relations between student engagement and learning performance. To evaluate the consistency of research papers, we coded the factors influencing student engagement. We then evaluated those measures using the same elements and used the elements to compile a composite score. 

### 3.4. Research Design

The study designs were coded as categorical variables, including whether the size of the effect of student engagement was derived from cross-sectional or longitudinal research (1 = cross-sectional, 2 = longitudinal). For the longitudinal studies, the effect sizes for measuring student engagement and the effect sizes for self-evaluation were also included. In addition, the size of the effect related to the influencing factors on student engagement was coded. Of the 55 eligible articles, 32% were randomly selected for double coding by the first two authors. The intraclass correlation (for continuous variables) and Cohen’s κ (for categorical variables) were calculated. In order to examine the factors influencing student engagement, the Pearson’s correlation coefficients were examined for all included studies. The zero-order correlation coefficients are bivariate estimates typically obtained from each empirical study’s correlation matrix or requested from the authors if none were provided in the full text. Further, to receive a similar direction of effects, we recoded effect sizes and applied Fisher’s r-to-z transformation (1921), converting the effect-size estimate from each association. 

### 3.5. Publication Bias

Several studies have shown that nonsignificant results are more likely to be rejected for publication. Thus, publication bias might cause inflated effect sizes and limited validity in meta-analyses. A statistical analysis of the possible impacts of publication bias should be considered before presenting final outcomes. This problem was addressed by using a funnel plot of the distribution of each individual study’s effect size on the horizontal axis against its precision, expressed in standard errors, on the vertical axis. Where a publication’s bias impacts the data, the asymmetrical funnel plot analyzes the significance of the asymmetry to offer more precise information on the possible presence of publication bias. Additionally, when this test yielded significant results, sensitivity analyses were conducted using the trim-and-fill method, correcting for the asymmetric plots by imputing missing effect sizes through several iterations. 

### 3.6. Data Analyses

All analyses were generated in the metafor package in the R software environment. The studies reported many effect sizes; for instance, studies with longitudinal data yielded effect sizes for various time points, and different raters resulted in multiple effect sizes for men and women. These different effect sizes from the same research were more similar than effect sizes from studies that did not share the same sample, data collection, and sampling methods. The multiple nested effect sizes were used to analyze the assumptions of meta-analyses. In addition, the dependency problem was applied to select effect sizes or neglect the dependency of effect sizes. Further, multilevel meta-analysis is considered an optimal tool to take dependency into account, including available effect sizes, maximum information, and statistical power. 

The current meta-analysis was generated in several stages: Overall mean effect sizes were estimated to assess the strength of the association between student engagement and learning outcomes in an educational context. The likelihood ratio test was then applied to evaluate the between-study and within-study heterogeneity. It is acknowledged that the level of study follows the databases. For the multilevel analyses, the same dataset allowed us to consider the dependency associated with the number of independent studies. Based on the heterogeneity in effect sizes, moderation analyses were generated with the conceptual interest and methodological features. The meta-analyses were generated based on the random-effects guidelines by applying the restricted maximum-likelihood procedure for parameter estimation. The mean correlation was analyzed by calculating the sample size in this study.

The meta-analysis is the synthesis of the analysis of existing research data and requires the extraction of literature information and research data through encoding and organizing the extracted information. An open coding method was explored for trial coding, and the coding scheme was constantly modified to ensure the development of a coding scheme suitable for the sample data of this study. The coding included content analysis of the literature, the research object, research content, conclusion extraction, and the effect of the covariance item. The coding work was conducted by two researchers at the same time, with a coding reliability of 95.6%. Those researchers coded the text independently and then reviewed the inconsistent parts of the original data, renegotiated, and finally obtained the coding text. The analysis focused mainly on sample size, and the correlation coefficient R was calculated according to the Fischer-Z transformation of t and *p* values. The analysis did not enable comparison of factors or repeated cases. In considering the many types of measurement results, the meta-analysis provides an average overview of comparable data and the most efficient results (see Table 1). 

## 4. Results

### 4.1. Overall Effect Value Analysis

The overall effect value analysis results of the meta-analysis are shown in Table 2, including the results of the heterogeneity test. Table 2 indicates that the Q-value of the overall effect value is 52596.610 (*p* < 0.001), which is much larger than the degree of freedom (*df* = 70), indicating that the effect value of each study is heterogeneous. Concurrently, I = 99.867, showing that the proportion of heterogeneity in the total variation of effect size is 99.867% (greater than 50%) and substantial heterogeneity exists. τ^2^ = 0.082, indicating that 8.2% of the variation between studies can be used to calculate the weight. In other words, the weight of each study assigned under the random effect model was 0.082. Therefore, the Q-value indicates significant heterogeneity in the overall effect value.

**Figure 3:**
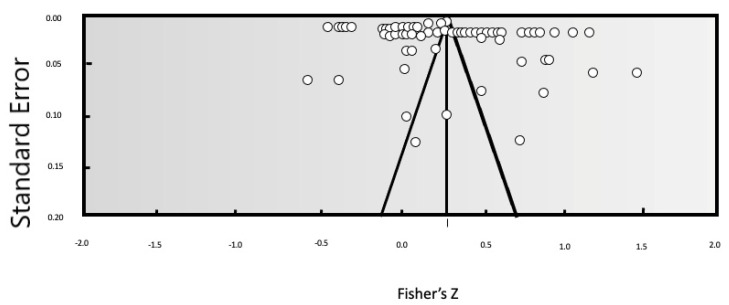
Funnel plot analysis of publication bias.

Evaluation of publication bias is a very important step in the component analysis method. There is a strong preference for high quality results by authors who have already published papers, and this means that the literature publication bias then leads to even further bias. The results of a meta-analysis may even lead to incorrect conclusions, reducing the value of the meta-analytic approach. Therefore, in the literature on meta-analyses, scholars typically use a combination of techniques to determine whether there is publication bias. In this paper, we combined a funnel plot, a loss of safety factor, and Egger’s regression intercept to make a reasonable and accurate judgment about bias. First, a qualitative analysis was carried out using a funnel plot of publication bias, shown in Figure 3. Most of the research results were concentrated at the top of the funnel plot, and few results appeared at the bottom of the plot. Moreover, the distribution of research points on both sides of the vertical line was relatively balanced, indicating that our meta-analysis has only a small possibility of publication bias. The coefficient of loss of safety was *N* = 3629 (*K* = 71), that is, 8404.6 negative experiments are required for each observed study to reverse the conclusion. The regression intercept of Egger was 4.0187, and the *p*-value was 0.4939 (>0.05), indicating that there was no significant difference between the intercept item and 0, and thus no publication bias.

### 4.2. Specific Effect Value Analysis

The heterogeneity was generally tested by Q and τ^2^ statistics. The heterogeneity test results of this study are shown in Table 3, where it can be seen that the results of the Q test were significant (all *p*-values < 0.05), indicating heterogeneity between studies. Meanwhile, the τ^2^ value of randomly selected influencing factors was greater than 80% and above 95%, demonstrating that the variation of each influencing factor accounts for a high proportion of the overall effect value. Therefore, the random effect model was used to analyze all the influencing factors in this study. However, we considered that *I*^2^ = 0% indicates no heterogeneity between studies. Mild heterogeneity is considered when *I*^2^ < = 25%; moderate heterogeneity when *I*^2^ < = 50%: and high heterogeneity when *I*^2^ < = 75%. *I*^2^ > 75% is not suitable for meta-analysis. 

### 4.3. Sensitivity Analysis and Publication Bias Analysis

This study undertook a multi-factor analysis. A sensitivity analysis was conducted on the assumption that the factors are independent of each other and revealed that the study has good stability. The publication bias analysis using Egger’s test indicated regression intercept *p*-values (*p*_1_ = 0.247, *p*_2_ = 0.493) greater than 0.05 with no significant difference between the intercept and 0. At the same time, the total loss of safety factor (*N* = 3629) of each influencing factor was much greater than the total value of K (*K* = 71). Thus, publication bias was not a problem for any influencing factor included in the meta-analysis.

### 4.4. Effect-Value Analysis

The results of the effect value analysis indicated that the learning background factor (*K* < 2) did not meet the screening criteria, i.e., that the independent variable factor must appear more than twice and could therefore be eliminated. In addition, the negative emotional factor (*K* < 2), with a confidence interval ranging from −0.234 to 0.273, failed the test and was also excluded. Students’ participation in learning activities is not affected by their learning background (e.g., previous learning experience or academic performance), and excellent academic performance does not mean that students have high participation in class. Further, students’ negative emotions in the learning process, such as weariness and burnout, did not significantly affect their learning participation (see Table 4).

## 5. Discussion

The meta-analysis revealed 14 factors affecting students’ learning participation. On the basis of the screening requirements in our summary results, we eliminated two factors, namely, learning background and students’ negative emotions. One factor was strongly correlated with students’ participation in the study; two factors had a moderate intensity of correlation; and 11 factors were weakly correlated with learning participation. The factors influencing students’ participation were relatively dispersed and did not show a highly unified expression paradigm. These varied influencing factors in the international empirical literature are a significant finding of this study [26]. 

Of these, the most influential factor for students’ participation in learning activities is individual positive emotion (*R* = 0.751, *p* < 0.001). This supports the conclusions of Arguedas and Daradoumis (2016) but is not consistent with scholars who suggest that positive teacher-student relationships and partner support are key to students’ participation in learning activities. Only two articles focused on students’ positive emotions as influencing factors, but literature suggests that there are many dimensions and multiple factors related to including students. These include the degree to which students are satisfied with the curriculum, the recognition of teachers, a classes and grade’s sense of belonging, and other positive emotions. Based on their own internal power source, students learn to participate, leading to direct and significant correlations. In addition, the reasons for low student engagement are varied and complex, involving a range of social, family, and personal factors: poverty, lack of purpose, poor physical and mental health, environmental and community factors, parental attitudes, etc. There is a clear link between these factors and low participation. However, discussions about low participation rarely highlight young people’s acceptance of what is being taught in school [27].

The external influencing factors with moderate correlations were the teacher-student relationship (*R* = 0.456, *p* < 0.001) and positive teacher behavior (*R* = 0.419, *p* < 0.001). These findings suggested that teacher-student relationships and positive teacher behavior are key factors affecting students’ participation in classroom learning. Researchers have mostly considered external influences and support. A good teacher-student relationship facilitates a relaxed and pleasant learning state for students, and positive teacher behaviors–such as guidance, motivation, timely feedback, and other supportive behaviors–all promote students’ learning participation. At present, the research results on relevant issues are scattered, and the translation of international empirical literature will be affected by expression [28].

The meta-analysis indicated that there are many influential factors that are weakly correlated with learning participation. The main external influencing factor was partnership (*R* = 0.174, *p* < 0.001). A good partnership will promote cooperative learning between learning communities and learning cooperative group participation is key to students’ participation in class. The learning resources (*R* = 0.128, *p* < 0.001) (e.g., the acceptability and readability of learning materials and the advanced and intuitive nature of classroom teaching technology) serve to support students’ learning activities and classroom participation. The teaching materials should be in line with students’ cognitive level, and vivid and intuitive multimedia technology will improve learning participation. The teaching factors (*R* = 0.009, *p* < 0.001) also affect students’ learning participation, e.g., clear teaching, conducive learning environments, and whether the teaching plan and curriculum arrangement meet the appropriate level. 

In environmental support (*R* = −0.028), negative teacher behavior (*R* = −0.064), and negative learning behavior (*R* = −0145) were all negatively correlated with learning participation. These findings are reflected in the fact that environmental support (e.g., curriculum and homework requirements) can create excessive pressure for students, negative emotions, and resistance. If teachers demonstrate negative behaviors, such as criticism and scolding, students will have negative emotions and learning behaviors, such as weariness, absenteeism, and emotional anxiety, and their learning participation will be reduced. There were several internal factors associated with a low degree of learning participation. The students’ personality characteristics (*R* = 0.218, *p* < 0.001) were linked to low participation. The students with open-minded personalities were more willing to actively participate in classroom learning activities. Other personality traits included students’ learning (*R* = 0.212) and thinking abilities (*R* = 0.199)—representing the basic abilities of students in the learning process. Students should not only “learn to learn” but also “learn to think.” Those with high knowledge acceptance, quick reactions, a willingness to think, and a strong thinking ability tend to participate more actively in classroom learning. They are also more likely to show positive learning behaviors (*R* = 0.187) such as actively answering questions, cooperating, listening carefully, and completing homework on time. These behaviors are all positive manifestations of students’ participation in learning activities, as summarized in Table 5.

The process of identifying and measuring student engagement is often fraught with difficulties. A number of studies have demonstrated that defining engagement is often confusing and overlapping. Nevertheless, some studies have emerged in recent years on the basis of the concept of student engagement. Three elements, initially viewed in isolation but more recently more comprehensively, have become widely accepted: Thinking/cognition, a feeling/mood/emotion, and performance/action/operation. The specific indicators are somewhat different, but there is agreement on models that identify and measure the way students think, feel, and behave at school. In addition to students, when the responsibility for participation falls on a wider range of partners, we are only likely to see deep, authentic, and passionate learning when all partners support a “how to” rather than a “how to” approach to learning. Therefore, it is important to shape not just engaged students but engaged schools [29,30].

## 6. Conclusions

This paper used the meta-analysis method to analyze students’ learning parameters and explore their influencing factors. The influencing factors were divided into two main categories factors that promote or hinder learning. The promoting factors included: students’ positive emotion, positive learning behavior, positive teacher behaviors, teacher-student relationship and partnership, students’ learning and thinking ability, support of learning resources, students’ individual and personality characteristics, and teaching factors. The hindering factors were environmental support, negative learning behavior, and negative teacher behavior. Further, a small set of factors were found to have no significant relationship with learning [31,32,33].The factors of learning background and negative emotion had no significant correlation and were eliminated following the meta-analysis screening criteria. Based on previous studies, we can conclude that there are many factors that affect student engagement, but there are only two categories. The first type refers to students’ internal factors, including ideas, methods, intellectual factors (attention, memory, thinking), and non-intellectual factors (learning motivation, learning interest, personality, emotion, learning attitude, learning habits, etc.). The second category refers to students’ external factors, including social education environment, family education environment, and school education environment in all three aspects. Among them, the non-intellectual factor of students is the key factor [34,35,36,37].

The research results provide an accurate estimate of the factors influencing students’ learning participation in international empirical literature. A summary of the typical influencing factors related to students’ learning participation is presented, providing an important supplement to current studies of learning participation. At the same time, the meta-analysis elucidates teaching research focusing on the promotion of students’ learning engagement and ways to improve classroom participation.

However, this study has some limitations. First, the literature included in the meta-analysis only contained the data required by the meta-analysis, and many research papers were excluded from the study because we could not obtain relevant data. This may have led to inaccurate conclusions because of incomplete data. Second, factors such as negative emotion are explicitly mentioned in the literature as obstructing students’ intention to participate in learning and behavior. However, there were fewer than two papers containing this factor. Thus, we could not include this factor in the analysis of this paper. In the future, differences in influencing factors on students’ learning participation at home and abroad should be studied further, together with the moderating variables, to derive more convincing universal conclusions.

## Figures and Tables

**Table 1:** A representative sample of partial literature coding of factors influencing student engagement.

No.	Author	Subjects (Sample Size)	Effect Value Statistics	Influencing Factors
2	Marta Arguedas	144	Sample size and correlation coefficient	Teacher-involvement (0.245) Teacher-structure (−0.015) Teacher-autonomy support (0.215)
3	Ellen A. Skinner	160	Sample size and correlation coefficient	Awareness-happiness (0.561) Awareness-sadness (−0.069) Awareness-fear (0.093) Awareness-anger (0.040)
6	George D. Kuh	80479	Sample size and correlation coefficient	Academic efforts (0.157) Higher order thinking (0.201) Academic integration (0.193) Active and collaborative learning (0.264)
4	Arnold B. Bakker Ana Isabel Sanz Vergel Jeroen Kuntze	4596	Sample size and T value	Intercept (40.513) Age (0.948) Following other studies (−1.559) Hours preparing tutorial group (2.066) Weekly study resources (3.970) Weekly personal resources (4.837) Trait openness (1.862) Personal resources × openness (4.461)
8	Angela Boatman Bridget Terry Long	5500	Sample size and correlation coefficient	Hours worked per week in college (0.936) Discussed ideas with faculty (0.060) Worked with other students (0.252) Participating in residence hall events (0.148) Participation in community service (0.140)
9	Robert M. Carini	1058	Sample size and correlation coefficient	Level of academic challenge (0.11) Active and collaborative learning (0.02) Student-faculty interaction (0.03) Enriching educational experiences (0.09) Supportive campus climate (0.08) Reading and writing (0.12) Quality of relationships (0.14) Institutional emphases on good practices (0.03) Higher-order thinking (0.08) Student-faculty interaction concerning coursework (0.03) Integration of diversity into coursework (0.10)
10	Julie A. Gray	187	Sample size and correlation coefficient	Learner interaction (0.652) Instructor presence (0.403)
11	Selim Gunuc Abdullah Kuzu	805	Sample size and correlation coefficient	Valuing (0.547) Sense of belonging (0.761) Cognitive engagement (0.721) Peer relationships (0.547) Relationships with the faculty (0.769) Behavioral engagement (0.617)
12	Giselle Bonet Barbara R. Walters	263	Sample size and correlation coefficient	Class delivery modality (−0.334) Grades (−0.507)

**Table 2:** Distribution of overall effect values and heterogeneity test results.

Model	Effect Size and 95% Interval				Test of Null (2-Tail)		Heterogeneity				Tau-Squared	
Model	Number Studies	Point Estimate	Lower Limit	Upper Limit	Z	*p*	Q	*df*	*p*	*I* ^2^	τ^2^	SE
Fixed	71	0.232	0.2229	0.234	194.077	0.000	52,596.610	70	0.000	99.867	0.082	0.025
Random effects	71	0.268	0.205	0.329	8.043	0.000						

**Table 3:** Partial results of heterogeneity test.

Influencing Factor	K	N	Heterogeneity (Q Test)			Tau-Squared			
			Q Value	*p* Value	*I* ^2^	τ^2^	SE	Variance	τ
Supportive environment	4	21328	3353.143	0.000	99.911	0.157	0.148	0.022	0.396
Partnership	6	22,146	469.546	0.000	98.935	0.031	0.027	0.001	0.175
Positive teacher behavior	18	155,010	14,363.689	0.000	99.882	0.093	0.033	0.001	0.304
Positive emotions	2	865	7.063	0.000	85.841	0.057	0.094	0.009	0.239
Active learning behavior	5	88,332	1603.613	0.000	99.751	0.109	0.122	0.015	0.331
Teaching factors	4	2090	35.221	0.000	91.482	0.018	0.020	0.000	0.135
Student-teacher relations	8	70,187	4291.603	0.000	99.837	0.071	0.041	0.002	0.267
Negative teacher behavior	4	30,270	849.464	0.000	99.647	0.028	0.023	0.001	0.168
Negative emotions	1	60	0.000	1.000	0.000	0.000	0.000	0.000	0.000
Negative learning behavior	5	34,866	435.921	0.000	99.082	0.012	0.009	0.000	0.111
Individual characteristics	3	5129	104.192	0.000	98.080	0.015	0.021	0.000	0.124
Personality Characteristics	3	5891	750.736	0.000	99.734	0.349	0.384	0.147	0.591
Thinking ability	2	81,537	15.909	0.000	93.714	0.007	0.011	0.000	0.085
Learning ability	3	91,537	210.427	0.000	99.050	0.011	0.014	0.000	0.103
Study background	1	1058	0.000	1.000	0.000	0.000	0.000	0.000	0.000
Learning Resource	2	4866	33.289	0.000	96.996	0.005	0.007	0.000	0.072
In total	71		52,596.610	0.000	99.867	0.082	0.025	0.001	0.286

**Table 4:** Specific results of meta-analysis on factors affecting students’ learning participation.

Influencing Factor	K	N	R Value	95% Confidence Interval		Two-Tailed Test	
				Lower Limit	Upper Limit	Z-Value	*p*-Value
**Positive emotions**	**2**	**865**	0.751	0.720	0.778	28.559	0.000
Student-teacher relation	8	70,187	0.456	0.450	0.462	130.292	0.000
Positive teacher behavior	18	155,010	0.419	0.415	0.423	181.105	0.000
Student personality Characteristics	3	5891	0.218	0.193	0.242	16.969	0.000
Student learning ability	3	91,537	0.212	0.206	0.219	65.252	0.000
Thinking ability of students	2	81,537	0.199	0.193	0.206	57.728	0.000
Active learning behavior	5	88,332	0.187	0.181	0.194	56.23	0.000
partnership	6	22,146	0.174	0.162	0.187	26.208	0.000
Learning Resource	2	4866	0.128	0.112	0.144	15.583	0.000
Study background	1	1058	0.090	0.030	0.149	2.931	0.003
Personality Characteristics	3	5129	0.040	0.024	0.056	4.922	0.000
Negative emotions	1	60	0.021	−0.234	0.273	0.159	0.874
Teaching factors	4	2090	0.009	0.017	0.035	0.676	0.000
Supportive environment	4	21,328	−0.028	−0.040	−0.017	−5.018	0.000
Negative teacher behavior	4	30,270	−0.064	−0.074	−0.055	−12.881	0.000
Negative learning behavior	5	34,866	−0.145	−0.154	−0.136	−30.765	0.000

**Table 5:** Correlation of influencing factors on students’ learning.

Correlation Coefficient	Influencing Factors	
**Strong correlation (*r* ≥ 0.5)**	External	
	Internal	Positive emotions
Medium correlation (0.3 ≤ *r* < 0.5)	External	Positive teacher behavior, teacher-student relationship
	Internal	
Weak correlation (0.10 ≤ *r* < 029)	External	Partnership, learning resource support, teaching factors, negative teacher behavior, negative learning behavior, environmental support
	Internal	Student personality characteristics, student learning ability, student thinking ability, positive learning behavior, student individual characteristics

## Data Availability

Not applicable.

## References

[1] Astin A.W. (1984). Student involvement: A developmental theory for higher education. J. Coll. Stud. Pers..

[2] Appleton J.J., Christenson S.L., Furlong M.J. (2008). Student Engagement with School: Critical Conceptual and Methodological Issues of the Construct. Psychol. Sch..

[3] Coates H. (2005). The Value of Student Engagement for Higher Education Quality Assurance. Qual. High. Educ..

[4] Arguedas M., Daradoumis T., Xhafa F. (2016). Analyzing How Emotion Awareness Influences Students’ Motivation, Engagement, Self-Regulation and Learning Outcome. Educ. Technol. Soc..

[5] Kahu E.R., Nelson K. (2018). Student engagement in the educational interface: Understanding the mechanisms of student success. High. Educ. Res. Dev..

[6] Kahu E.R. (2013). Framing student engagement in higher education. Stud. High. Educ..

[7] Kahu E.R., Stephens C.V., Leach L., Zepke N. (2015). Linking academic emotions and student engagement: Mature-aged distance students’ transition to university. J. Furth. High. Educ..

[8] Klem A.M., Connell J.P. (2004). Relationships matter: Linking teacher support to student engagement and achievement. J. Sch. Health.

[9] Kuh G.D. (2001). Assessing what really matters to student learning: Inside the National Survey of Student Engagement. Change.

[10] Kuh G.D., Hayek J.C., Carini R.M., Ouimet J.A., Gonyea R.M., Kennedy J. (2001). NSSE Technical and Norms Report.

[11] Kuh G.D., Hu S. (2001). Learning productivity at research universities. J. High. Educ..

[12] Kuh G.D., Cruce T.M., Shoup R., Kinzie J., Gonyea R.M. (2008). Unmasking the effects of student engagement on first-year college grades and persistence. J. High. Educ..

[13] Kuh G.D. (2003). What we’re learning about student engagement from NSSE: Benchmarks for effective educational practices. Change Mag. High. Learn..

[14] Pike G.R., Kuh G.D. (2005). First and Second-generation College Students: A Com- parison of their Engagement and Intellectual Development. J. High. Educ..

[15] Pike G.R., Kuh G.D., Gonyea R.M. (2003). The Relationship between Institutional Mission and Student’s Involvement and Educational Outcomes. Res. High. Educ..

[16] Skinner E.A., Belmont M.J. (1993). Motivation in the classroom: Reciprocal effects of teacher behavior and student engagement across the school year. J. Educ. Psychol..

[17] Henrie C.R., Bodily R., Manwaring K.C., Graham C.R. (2015). Exploring intensive longitudinal measures of student engagement in blended learning. Int. Rev. Res. Open Distrib. Learn..

[18] Tan M., Hew K.F. (2016). Incorporating meaningful gamification in a blended learning research methods class: Examining student learning, engagement, and affective outcomes. Australas. J. Educ. Technol..

[19] Zhao C.M., Kuh G.D. (2004). Adding value: Learning communities and student engagement. Res. High. Educ..

[20] Bakker A.B., Vergel AI S., Kuntze J. (2015). Student engagement and performance: A weekly diary study on the role of openness. Motiv. Emot..

[21] Boatman A., Long B.T. (2016). Does Financial Aid Impact College Student Engagement?. Res. High. Educ..

[22] Carini R.M., Kuh G.D., Klein S.P. (2006). Student engagement and student learning: Testing the linkages. Res. High. Educ..

[23] Gray J.A., DiLoreto M. (2016). The effects of student engagement, student satisfaction, and perceived learning in online learning environments. Int. J. Educ. Leadersh. Prep..

[24] Gunuc S., Kuzu A. (2015). Student engagement scale: Development, reliability and validity. Assess. Eval. High. Educ..

[25] Bonet G., Walters B.R. (2016). High impact practices: Student engagement and retention. Coll. Stud. J..

[26] Masika R., Jones J. (2016). Building student belonging and engagement: Insights into higher education students’ experiences of participating and learning together. Teach. High. Educ..

[27] Quin D. (2017). Longitudinal and contextual associations between teacher–student relationships and student engagement: A systematic review. Rev. Educ. Res..

[28] Shernoff D.J., Kelly S., Tonks S.M., Anderson B., Cavanagh R.F., Sinha S., Abdi B. (2016). Student engagement as a function of environmental complexity in high school classrooms. Learn. Instr..

[29] You J.W. (2016). The relationship among college students’ psychological capital, learning empowerment, and engagement. Learn. Individ. Differ..

[30] Saritepeci M., Cakir H. (2015). The effect of blended learning environments on student motivation and student engagement: A study on social studies course. Egit. Bilim.

[31] Loveys B.R., Riggs K.M. (2019). Flipping the laboratory: Improving student engagement and learning outcomes in second year science courses. Int. J. Sci. Educ..

[32] Zepke N. (2018). Student engagement in neo-liberal times: What is missing?. High. Educ. Res. Dev..

[33] Gray C.C., Perkins D. (2019). Utilizing early engagement and machine learning to predict student outcomes. Comput. Educ..

[34] Tinto V. (2014). Reflective practice: Tinto’s South Africa lectures. J. Stud. Aff. Afr..

[35] Birch S., Ladd G. (1997). The teacher–child relationship and children’s early school adjustment. J. Sch. Psychol..

[36] Finn J.D., Pannozzo G.M., Voelkl K.E. (1995). Disruptive and inattentive withdrawn behavior and achievement among fourth graders. Elem. Sch. J..

[37] Li J., Xue E. (2022). Investigating international students’ cultivation system for higher education sustainability in China: Stakeholders’ perspective. Sustainability.

